# Sarcopenia, its components and fracture risk in people living with human immunodeficiency virus

**DOI:** 10.1590/1806-9282.20252134

**Published:** 2026-09-28

**Authors:** Edivaldo Cangussu Meira, Jaine Alves Ximenez, Marcia Midori Shinzato, Maria Claudia Bernardes Spexoto

**Affiliations:** 1 Universidade Federal da Grande Dourados, Faculty of Health Sciences – Dourados (MS), Brazil.; 2 Universidade Federal da Grande Dourados, Faculty of Health Sciences, Graduate Program in Food, Nutrition and Health – Dourados (MS), Brazil.

**Keywords:** HIV, Sarcopenia, Bone fractures, Muscle strength

## Abstract

**OBJECTIVE:**

The aim of this study was to evaluate the relationship between sarcopenia and its components with fracture risk in people living with human immunodeficiency virus, and to assess the association with sociodemographic and clinical characteristics.

**METHODS:**

This cross-sectional study was conducted with people living with human immunodeficiency virus undergoing antiretroviral therapy in the state of Mato Grosso do Sul, Brazil. Sarcopenia was defined according to the European Working Group on Sarcopenia in Older People criteria, assessing muscle strength (handgrip strength), skeletal muscle mass (skeletal muscle mass index), and physical performance (gait speed). The 10-year risk of major osteoporotic and hip fracture was estimated using the Brazilian version of the Fracture Risk Assessment Tool (FRAX^®^). Statistical analyses included chi-square tests, analysis of variance, and Spearman correlation (p<0.05).

**RESULTS:**

Among 105 participants (44.6±13.5 years), the prevalence of confirmed or severe sarcopenia was 6.7%. No association was found between sarcopenia and the risk of major fracture (p=0.471) or hip fracture (p=0.254). However, handgrip strength was correlated with the risk of major fracture (ρ=-0.21; p=0.029) and hip fracture (ρ=-0.25; p=0.010). SMII showed moderate correlations with major fracture risk (ρ=-0.44; p<0.01) and hip fracture risk (ρ=-0.52; p<0.01). Gait speed was correlated only with hip fracture risk (ρ=-0.22; p=0.025). Older age (p<0.001) and lower body mass index (p=0.011) were significant risk factors.

**CONCLUSION:**

Sarcopenia as a categorical diagnosis was not associated with fracture risk, whereas lower handgrip strength and skeletal muscle mass index were associated with higher FRAX^®^ estimates, and gait speed only with hip fracture risk. These findings support assessing sarcopenia-related parameters in people living with human immunodeficiency virus.

## INTRODUCTION

In the global scenario of 2023, 1.3 million new human immunodeficiency virus (HIV) infections were recorded. In Brazil, the detection rate was 17.1 cases per 100,000 inhabitants, contrasting with 22.4 in the state of Mato Grosso do Sul, and even higher rates in the municipality where this study was conducted^1^. Although notification is effective, the increased longevity achieved imposes challenges related to musculoskeletal health.

Sarcopenia, defined by the simultaneous presence of low muscle strength and low muscle mass^2^, is prevalent among people living with HIV (PLHIV), a population that experiences accelerated aging^3^ often accompanied by frailty^4^. Persistent chronic inflammation^5^, combined with the adverse effects of antiretroviral therapy (ART) on musculoskeletal health^6^, induces changes in body composition and reductions in bone mineral density (BMD)^7^. Specifically, the use of tenofovir disoproxil fumarate (TDF) is associated with impaired bone metabolism and the exacerbation of sarcopenia^7^.

The cumulative effects of these factors, combined with traditional risk factors, significantly increase the risk of fractures in PLHIV, especially among older individuals^8^. In this context, the use of tools such as FRAX^®^ (Fracture Risk Assessment Tool) becomes essential for risk stratification^9^. The present study aimed to evaluate the relationship between sarcopenia and its components (muscle strength, skeletal muscle mass, and physical performance) with fracture risk in PLHIV, as well as to assess the association with sociodemographic and clinical characteristics of this population.

## METHODS

### Design, population, and data collection

This cross-sectional observational study (Strengthening the Reporting of Observational Studies in Epidemiology guidelines—Strengthening the Reporting of Observational Studies in Epidemiology) was conducted in Dourados, MS, Brazil (April 2023 to December 2024) at the University Hospital of the Federal University of Grande Dourados/Hospital Universitário da Universidade Federal da Grande Dourados (HU-UFGD) and at the Specialized Care Service and Testing and Counseling Center (SAE/CTA). Participants were recruited consecutively among eligible PLHIV receiving care in outpatient and inpatient settings during the study period. Eligible individuals were those on ART who were able to undergo the study assessments and provide informed consent. Individuals with complete mobility restrictions, cognitive impairment preventing consent, edema, or an HIV diagnosis established during the hospitalization in which study recruitment occurred were excluded.

Data collection was carried out through the administration of structured forms and the review of medical records (both physical and electronic). Sociodemographic and clinical variables were assessed. The sociodemographic variables included age (years), sex, race/skin color, marital status, and educational level. The clinical variables comprised disease status (asymptomatic, symptomatic, or Aids), history of opportunistic and chronic diseases (yes/no), body mass index (BMI) in kg/m^2^, CD4+ count (>200 or ≤200 cells/mm^3^), and ART regimens.

### Assessment of sarcopenia and its components

Sarcopenia was defined according to the revised criteria of the European Working Group on Sarcopenia in Older People (EWGSOP2)^2^. Muscle strength was assessed using handgrip strength (HGS), measured with a hydraulic dynamometer (SAEHAN^®^, model SH5001), considering the highest value among three trials (cutoff points: <27 kg for men; <16 kg for women). Skeletal muscle mass (SMM) was estimated using the Lee equation^10^ and divided by height squared to calculate the skeletal muscle mass index (SMMI), adopting cutoff points established for the Brazilian population (men <8.9 kg/m^2^; women <6.4 kg/m_2_)_11_. Physical performance was evaluated using gait speed (GS), with a cutoff value of ≤0.8 m/s^2^. Participants were categorized as non-sarcopenic, probable sarcopenia, confirmed sarcopenia, or severe sarcopenia, according to the EWGSOP2 algorithm^2^. In this study, confirmed sarcopenia and severe sarcopenia were grouped together and classified as "sarcopenia."

### Fracture risk

The 10-year risks of major osteoporotic fracture (MOF) and hip fracture (HF) were estimated using the FRAX^®^ Brazil tool and expressed as percentages^12^. FRAX^®^ estimates fracture probability based on clinical risk factors, with or without BMD. For descriptive comparisons, FRAX^®^ estimates were categorized according to their distribution in the sample into three quantile-based groups, labeled Q1, Q2, and Q3. For MOF, the categories were Q1 (≤0.40%), Q2 (>0.40% to ≤1.00%), and Q3 (>1.00%). For HF, the categories were Q1 (0.00%), Q2 (>0.00% to ≤0.20%), and Q3 (>0.20%).

### Statistical analysis

The analyses were performed using Statistical Package for the Social Sciences version 22. Normality was assessed using the Kolmogorov-Smirnov test. Descriptive statistics were applied, and Pearson's chi-square or Fisher's exact tests were used to examine associations, while analysis of variance (ANOVA) was employed for multiple comparisons. The correlation between sarcopenia components and FRAX^®^ scores was evaluated using Spearman's coefficient (ρ). A significance level of p<0.05 was adopted.

### Ethical considerations

The study was approved by the Research Ethics Committee of UFGD under approval numbers 5.919.928; 6.559.968; and 7.684.435. All participants signed the informed consent form.

## RESULTS

A total of 105 participants (81 outpatients and 24 inpatients) were evaluated after the exclusion of 12 individuals based on clinical criteria and 14 refusals. The sample had a mean age of 44.6±13.6 years, with a slight predominance of males (51.4%). Sociodemographic characteristics and their association with fracture risk are detailed in Table 1. Age was significantly associated with both MOF and HF FRAX^®^ estimates (p<0.001 for both), with participants in Q3 being older.

**Table 1:** Sociodemographic variables and their relationship with fracture risk (Dourados, MS, Brazil, 2023–2024).

Variables		n*	Major osteoporotic fracture risk, FRAX^®^ (%)					Hip fracture risk, FRAX^®^ (%)			
			%†	Q1‡	Q2§	Q3||	p¶ value	Q1‡	Q2§	Q3||	p¶ value
Age (years) (Mean±SD**)		44.6±13.6		34.8±7.5	41.8±12.2	51.1±13.2	<0.001	35.2±7.8	44.9±11.5	51.1±14.6	<0.001
Sex											
	Male	54	51.4	14	16	24	0.439	15	20	19	0.410
	Female	51	48.6	13	10	28		15	13	23	
Self-reported race/skin color											
	Non-white	75	71.4	19	21	35	0.459	22	24	29	0.906
	White	30	28.6	8	5	17		8	9	13	
Marital status											
	Married	34	32.4	8	11	15	0.122	10	13	11	0.201
	Single	46	43.8	14	9	23		15	12	19	
	Widowed	7	6.7	-	-	7		-	1	6	
	Separated/divorced	18	17.1	5	6	7		5	7	6	
Education level											
	<4 years	11	10.5	2	3	6	0.050	2	4	5	0.523
	4 to 8 years	44	41.9	12	4	28		12	10	22	
	8 to 11 years	29	27.6	8	12	9		10	11	8	
	>11 years	21	20.0	5	7	9		6	8	7	

* n: sample size

† %: percentage

‡ Q1

§ Q2, and

|| Q3: quantile-based FRAX^®^ risk groups according to the sample distribution, expressed as 10-year fracture probabilities (%)

¶ p: Pearson's chi-square test was used for categorical variables, and the ANOVA (analysis of variance) test was used for the numerical variable

** SD: standard deviation; FRAX^®^: Fracture Risk Assessment Tool. Bold values indicate statistical significance (p<0.05).

Regarding clinical characteristics (Table 2), the mean BMI was 24.19±6.20 kg/m^2^ and was associated only with HF FRAX^®^ estimates (p=0.011), which participants in Q3 showed lower BMI values.

**Table 2:** Clinical variables and their relationship with fracture risk (Dourados, MS, Brazil, 2023–2024).

Variables		n*	%†	Major osteoporotic fracture risk, FRAX^®^ (%)			p¶ value	Hip fracture risk, FRAX^®^ (%)			p¶ value
				Q1‡	Q2§	Q3||		Q1‡	Q2§	Q3||	
Disease status											
	Asymptomatic	32	30.5	12	6	14	0.384	13	8	11	0.332
	Symptomatic	37	35.2	7	9	21		8	11	18	
	Aids	36	34.3	8	11	17		9	14	13	
Opportunistic diseases											
	Absent	69	65.7	19	19	31	0.418	21	22	26	0.768
	Present	36	34.3	8	7	21		9	11	16	
Previous chronic diseases											
	Absent	70	66.7	17	18	35	0.881	19	22	29	0.879
	Present	35	33.3	10	8	17		11	11	13	
	Body mass index¶ (kg/m^2^) (Mean±SD**)	24.19±6.20		25.6±5.3	24.7±7.8	23.2±5.7	0.257	26.2±5.6	25.1±7.0	22.1±5.3	0.011
	CD4+††† (cells/mm^3^)‡‡‡ (Med§§§)	342.5					0.588				0.491
	≤200 cells/mm^3^	36	34.3	8	11	17		9	14	13	
	>200 cells/mm^3^	69	65.7	19	15	35		21	19	29	

* n: sample size

† %: percentage

‡ Q1

§ Q2, and

|| Q3: quantile-based FRAX^®^ risk groups according to the sample distribution, expressed as 10-year fracture probabilities (%)

¶ p: Pearson's chi-square test was used for categorical variables, and the ANOVA (analysis of variance) test was used for the numerical variable

** SD: standard deviation; FRAX^®^: Fracture Risk Assessment Tool

††† CD4+: CD4+ T-helper lymphocyte count

‡‡‡ 2 missing cases

§§§ Med: median. Bold values indicate statistical significance (p<0.05).

TDF was used by 75.2% of the participants. The predominant ART regimen was a triple therapy consisting of two nucleos(t)ide reverse transcriptase inhibitors and one integrase inhibitor (66.7%).

The distribution of participants according to sarcopenia status was 78.1% non-sarcopenic, 15.2% probable sarcopenia, and 6.7% confirmed or severe sarcopenia. Sarcopenia as a categorical diagnosis was not associated with MOF (p=0.471) or HF risk estimates (p=0.254). In contrast, when sarcopenia-related parameters were analyzed individually, SMMI was inversely correlated with MOF (ρ=-0.44; p<0.01) and HF (ρ=-0.52; p<0.01), and HGS was also inversely correlated with MOF (ρ=-0.21; p=0.029) and HF (ρ=-0.25; p=0.010). GS was inversely correlated only with HF risk (ρ=-0.22; p=0.025) (Table 3).

**Table 3:** Correlation between sarcopenia components and fracture risk scores among participants (Dourados, MS, Brazil, 2023–2024).

Components of sarcopenia		Major fracture risk	Hip fracture risk
HGS*	ρ†	-0.21	-0.25
	p‡	<0.029	<0.010
SMII§	ρ†	-0.44	-0.52
	p‡	<0.010	<0.010
GS||	ρ†	-0.16	-0.22
	p‡	0.095	<0.025

* HGS: maximum handgrip strength

† ρ: Spearman correlation coefficient

‡ p: significance level

§ SMII: skeletal muscle mass index

|| GS: gait speed.

## DISCUSSION

In this study, sarcopenia as a categorical diagnosis was not associated with MOF or HF risk FRAX^®^ estimates. In contrast, lower SMMI and HGS were correlated with higher MOF and HF estimates, whereas lower GS was correlated only with HF. This pattern may reflect the low prevalence of confirmed/severe sarcopenia and the loss of information when continuous measures are categorized into a composite diagnosis. Older age was associated with both outcomes, and lower BMI with HF.

The lack of an association with sarcopenia in our study contrasts with evidence from the literature. In one cohort, individuals with sarcopenia showed a significantly higher risk of osteoporotic fracture (HR 2.11 [95%CI 1.47–3.04]), with this risk being even higher among women (HR 2.40 [95%CI 1.51–3.81])^13^. Supporting this scenario, recent data indicate that osteosarcopenia—defined as the coexistence of sarcopenia and osteopenia—(with a global prevalence of 18.5%) represents a strong risk factor for fractures (HR 2.13), falls, and mortality^14^. This divergence in our findings may reflect the sample size or specific clinical characteristics of the population studied, which presented advanced immunosuppression (median CD4+ count of 342.5 cells/mm^3^). The literature indicates that HIV infection and chronic inflammation dysregulate the RANKL (receptor activator of nuclear factor κB ligand)/OPG (osteoprotegerin) pathway, enhancing osteoclastic activity and accelerating bone loss^15^. In addition, the predominant use of TDF in the sample is a well-known factor associated with reduced BMD^5^.

Low BMI was significantly associated with HF risk, supporting evidence that low body weight is an independent risk factor, reflecting reduced muscle and bone reserves^16^. Although the use of integrase strand transfer inhibitors (present in the ART regimen of most PLHIV in this sample) has been associated with weight gain in a systematic review^17^, maintaining an adequate BMI remains a critical protective factor.

The analysis of sarcopenia components revealed that muscle strength and muscle mass are relevant indicators. Low HGS emerged as a risk predictor, aligning with recent studies that position it as an effective tool for screening bone fragility, independent of BMD^18^. Similarly, GS was correlated with HF risk; evidence suggests that slow GS may increase fracture risk by up to 1.8-fold^16^, whereas preserved mobility serves as a protective factor against sarcopenia in PLHIV^19^.

Early aging, exacerbated by the interaction between HIV, ART, and comorbidities, accelerates musculoskeletal deterioration^20^, which justifies the association observed between older age and fractures.

The main limitations of this study are its cross-sectional design, modest sample size, and inclusion of participants from both outpatient and inpatient settings. Although this approach reflects real-world care, these settings may differ in disease severity, mobility, nutritional status, and muscle function. Given the small number of inpatients, stratified analyses by care setting were not performed. Accordingly, the findings should be interpreted as applying to the overall study population rather than to each care setting separately.

As strengths, the study demonstrated the relevance of sarcopenia components in functional assessment for fracture risk. Moreover, the use of validated tools (EWGSOP2, FRAX^®^) in an understudied population supports the implementation of multidisciplinary screening routines in PLHIV.

## CONCLUSION

Although categorical sarcopenia was not associated with FRAX^®^ estimates, lower HGS and SMMI were correlated with higher MOF and HF estimates, while lower GS and BMI were correlated only with HF. Older age was associated with both outcomes. These findings support the assessment of sarcopenia-related parameters in PLHIV.

## ETHICAL APPROVAL

The study was approved by the Research Ethics Committee of the Federal University of Grande Dourados (UFGD) on March 1, 2023, under approval numbers 5.919.928, 6.559.968, and 7.684.435, and all participants signed the informed consent form.

## Data Availability

The datasets generated and/or analyzed during the current study are available from the corresponding author upon reasonable request.

## References

[1] UNAIDS (2024). The urgency of now: AIDS at a crossroads. Global AIDS update 2024.

[2] Cruz-Jentoft AJ, Bahat G, Bauer J, Boirie Y, Bruyère O, Cederholm T (2019). Sarcopenia: revised European consensus on definition and diagnosis. Age Ageing.

[3] Schrock JM (2024;). Accelerated aging in people living with HIV: the neuroimmune feedback model. Brain Behav Immun Health.

[4] Tsakona D, Kapetanakis A, Kyrou D, Cholongitas E, Deutsch M, Malandris K (2023). Mapping frailty in people living with HIV: a nationwide study in Greece. HIV Med.

[5] Mu W, Patankar V, Kitchen S, Zhen A (2024). Examining chronic inflammation, immune metabolism, and T cell dysfunction in HIV infection. Viruses.

[6] Brañas F, Torralba M, Antela A, Vergas J, Ramírez M, Ryan P (2023). Effects of frailty, geriatric syndromes, and comorbidity on mortality and quality of life in older adults with HIV. BMC Geriatr.

[7] McGee DM, Cotter AG (2024). HIV and fracture: risk, assessment and intervention. HIV Med.

[8] Lerner ED, Mahmud IC, Schneider RH (2023). Mortality and osteoporosis in older adults living with HIV/AIDS: a systematic review. Rev Amaz Geriatr Gerontol.

[9] Mazzitelli M, Branca IP, Muramatsu T, Carli L, Dusina A, Focà E (2022). FRAX assessment in people ageing with HIV. HIV Med.

[10] Lee RC, Wang Z, Heo M, Ross R, Janssen I, Heymsfield SB (2000). Total-body skeletal muscle mass: development and cross-validation of anthropometric prediction models. Am J Clin Nutr.

[11] Alexandre TS, Duarte YA, Santos JL, Wong R, Lebrão ML (2014). Prevalence and associated factors of sarcopenia among elderly in Brazil: findings from the SABE study. J Nutr Health Aging.

[12] Zerbini CA, Szejnfeld VL, Abergaria BH, McCloskey EV, Johansson H, Kanis JA (2015;). Incidence of hip fracture in Brazil and the development of a FRAX model. Arch Osteoporos.

[13] Zhang HW, Tsai ZR, Chen KT, Lin CC, Lin HY, Chen YM (2022). Enhanced risk of osteoporotic fracture in patients with sarcopenia: a national population-based study in Taiwan. J Pers Med.

[14] Chen S, Xu X, Gong H, Chen R, Guan L, Yan X (2024). Global epidemiological features and impact of osteosarcopenia: a comprehensive meta-analysis and systematic review. J Cachexia Sarcopenia Muscle.

[15] Ahmed M, Mital D, Abubaker NE, Panourgia M, Owles H, Papadaki I (2023). Bone health in people living with HIV/AIDS: an update of where we are and potential future strategies. Microorganisms.

[16] Azevedo DC, Hoff LS, Kowalski SC, Andrade CAF, Trevisani VFM, Melo AKG (2024;). Risk factors for osteoporotic hip fracture among community-dwelling older adults: a real-world evidence study. Adv Rheumatol.

[17] Kanters S, Renaud F, Rangaraj A, Zhang K, Limbrick-Oldfield E, Hughes M (2022;). Evidence synthesis evaluating body weight gain among people treating HIV with antiretroviral therapy - a systematic literature review and network meta-analysis. EClinicalMedicine.

[18] Huang SG, Lee RP, Yao TK, Wang JH, Wu WT, Yeh KT (2025). Correlation between handgrip strength and bone density and fragility fracture risk among older adults: a cross-sectional study. J Nurs Res.

[19] SeyedAlinaghi S, Ghayomzadeh M, Mirzapour P, Maroufi SF, Pashaei Z, Ali Z (2023). A systematic review of sarcopenia prevalence and associated factors in people living with human immunodeficiency virus. J Cachexia Sarcopenia Muscle.

[20] Huang T, Li C, Chen F, Zhang Y, Liu Y, Li L (2023). Prevalence and risk factors of osteosarcopenia: a systematic review and meta-analysis. BMC Geriatr.

